# Severe hypercalcemia caused by repeated mineral oil injections: a case report

**DOI:** 10.20945/2359-3997000000591

**Published:** 2023-01-25

**Authors:** Raissa Carneiro Rezende, Isabella Carvalho Oliveira, Dandara Sampaio Leão de Carvalho, Guilherme Borges Andrade, Ana Beatriz Marinho de Jesus Teixeira, Whemberton Martins de Araújo, Monike Lourenço Dias Rodrigues

**Affiliations:** 1 Universidade Federal de Goiás Divisão de Endocrinologia Departamento de Medicina Interna Goiânia GO Brasil Universidade Federal de Goiás, Departamento de Medicina Interna, Divisão de Endocrinologia, Goiânia, GO, Brasil.; 2 Centro de Diagnóstico por Imagem Departamento de Medicina Nuclear Goiânia GO Brasil Centro de Diagnóstico por Imagem (CDI), Departamento de Medicina Nuclear, Goiânia, GO, Brasil.

## Abstract

Hypercalcemia is a frequent condition in clinical practice and when the most frequent causes are excluded, etiological diagnosis can be challenging. A rare cause of PTH-independent hypercalcemia is described in the present case report. A male adult with a history of androgenic-anabolic steroids abuse, and injection of mineral oil and oily veterinary compound containing vitamins A, D and E into muscles for local hypertrophy presented with hypercalcemia, nephrocalcinosis, and end-stage renal disease. On physical examination, the presence of calcified subcutaneous nodules and calcification of musculature previously infused with oily substances drew attention. Laboratory tests confirmed hypercalcemia of 12.62 mg/L, low levels of PTH (10 pg/mL), hyperphosphatemia (6.0 mg/dL), 25(OH)D of 23.3 ng/mL, and elevated 1,25(OH)2D (138 pg/mL). Imaging exams showed diffuse calcification of muscle tissue, subcutaneous tissue, and organs such as the heart, lung, and kidneys. The patient was diagnosed with PTH-independent hypercalcemia secondary to foreign body reaction in areas of oil injection. The patient underwent treatment with hydrocortisone for 10 days, single dose zoledronic acid and hemodialysis. He evolved with serum calcium levels of 10.4 mg/dL and phosphorus of 7.1 mg/dL. In addition, sertraline and quetiapine were prescribed to control body dysmorphic disorder. The medical community should become aware of new causes of hypercalcemia as secondary to oil injection since this should become increasingly frequent due to the regularity with which such procedures have been performed.

## INTRODUCTION

Hypercalcemia is a challenging condition in clinical practice, accounting for approximately 0.6% of all acute medical admissions (1). The most common etiologies are hyperparathyroidism and malignancy, corresponding to 90% of all cases (2,3). Frequently presents as asymptomatic biochemical testing but can require emergency management when severe (12 to 14 mg/ dL) (4). Clinical manifestations affect the neuromuscular, gastrointestinal, renal, skeletal, and cardiovascular systems (4). For diagnostic and treatment purposes, it can be classified as PTH- dependent hypercalcemia, when PTH (parathyroid hormone) levels are increased, and PTH-independent hypercalcemia, when PTH levels are normal or decreased (2).

When the most frequent causes are excluded, etiological diagnosis can be challenging. Vitamin D-associated [25(OH) or 1,25(OH)] hypercalcemia must be investigated in patients with low PTH levels. It might be a relatively uncommon etiology, but its prevalence is increasing due to indiscriminate vitamin D supplementation and new information becoming available on the prevalence of CYP24A1 mutations (2).

Vitamin D-mediated hypercalcemia can be caused due to exogenous vitamin D toxicity when excessive amounts of vitamin D metabolites or analogs are administrated; excessive production of 1,25(OH)D, as in granulomatous diseases such as tuberculosis or sarcoidosis and malignant lymphoproliferative diseases; and mutations in enzymes associated with vitamin D metabolite degradation (2).

In recent years, with the increasing abuse of injectable fillers for cosmetic purposes, hypercalcemia associated with cosmetic injections has been described (5,6). These fillers are often used for cosmetic augmentation of the breast and buttocks (7), but there are also a few case reports describing hypercalcemia associated with intramuscular injections of paraffin oil, mineral oil, or oily veterinary supplements to augment muscle size in bodybuilders (8-12). This new etiology fits in the role of granulomatous causes of hypercalcemia, as a foreign body reaction with granuloma formation expressing 1 α-hydroxylase in areas injected with body fillers has been described (8).

Despite uncommon, vitamin-D mediated hypercalcemia should be investigated in patients with unclear etiology, including calcitriol dosage, as delay in diagnosis and treatment can eventually lead to severe complications, such as cardiac arrhythmias or renal failure (2). Therefore, this paper aims to report the case of a young male chronic user of androgenic-anabolic steroids and mineral oil injections for aesthetic purposes that evolved with hypercalcemia, diffuse calcinoses, and dialytic renal failure. This study was conducted under the principles of the Declaration of Helsinki and was approved by the Research Ethics Committee of the Clinical Hospital of the Federal University of Goiás (Certificate of Presentation for Ethical Appreciation approval number 55601321.7.0000.5078), which waived the need for informed consent by the participant.

## CASE

A 27-year-old white male presenting with recurrent symptomatic hypercalcemia was admitted to the nephrology department for hospitalization. The patient referred asthenia, frequent vomiting, weight loss, dyspnea, dry cough, and, more recently, left audition acuity reduction and right hemiface peripheral paralysis. Over the most recent 4 years, there were multiple hospitalizations for nephrolithiasis attacks, urinary tract infection and obstruction, and double j catheter implant. Eighteen months ago, he evolved with renal failure and hemodialysis.

The patient had received multiple mineral oil and oily veterinary product composed of vitamins A, D, and E intramuscular injections in the biceps, triceps, back, and chest for aesthetic purposes for several years. He reported having stopped the practice 2 years ago. The oil injections were complicated with muscle hardening and calcified subcutaneous nodules.

His past medical history was significant for cocaine and androgenic-anabolic steroids (stanozolol, testosterone, nandrolone, and oxymetholone) abuse, systemic arterial hypertension, and anxiety disorder. The patient was on amlodipine, losartan, and clonidine in maximum doses for pressure control. He had no family history of parathyroid disorders or renal stones.

On physical examination, there were diffuse palpable calcified skin nodules and many warm, dense nodules in the subcutaneous tissue overlying the pectoral, trapezius, and biceps muscles. His blood pressure was 120/90 mmHg. There was no peripheral edema. The lungs, heart, and abdominal examinations were normal. No neurologic deficits other than peripheral facial paralysis were detected.

Blood chemistries at the initial evaluation revealed albumin-corrected calcium of 12.62 mg/dL (range, 8.3 to 10.6 mg/dL), and previous dosages of up to 15.9 mg/L, high levels of phosphorus, creatinine, and urea. The viral serologies for hepatitis B, C, and HIV were negative. Basal cortisol levels, thyroid function, total testosterone, luteinizing hormone, follicle-stimulating hormone, growth hormone, and prolactin showed no significant changes. Intact-PTH levels were reduced for renal insufficiency stage 5 CKD (13), 25-hydroxyvitamin D [25(OH)D] was low, and 1,25(OH)2D was elevated (Table 1).

**Table 1:** Laboratory results

Laboratory parameters	1.5 year pre-admission	1 year pre-admission	Patient admission	Patient discharge
Urea (RV 19-49 mg/dL)	190	157	139	129
Creatinine (RV 0.5-1.1 mg/dL)	12.3	11.58	8.8	7.4
Sodium (RV 136-145 mEq/L)	135	135	140	138
Potassium (RV 3.5-5.1 mEq/L)	3.5	6.6	4.1	5.3
CalciumCr (RV 8.3-10.6 mg/dL)	10.38	15.94	12.62	10.4
Phosphor (RV 2.5-4.5 mg/dL)	8.6	8.3	6.0	7.1
Parathyroid hormone (RV 18.5-88 pg/mL)	15	11.1	10	-
25(OH) vitamin D (RV > 20 ng/mL)	15.2	22.9	23.3	-
1,25(OH)_2_ vitamin D (RV 19.9-79.3 pg/mL)	-	109	138	-
Thyroid stimulating hormone (RV 0.35-4.94 μIU/mL)	-	-	-	1.25
Free thyroxine (RV 0.7-1.48 ng/dL)	-	-	-	0.82
Total testosterone (RV 166-877 ng/dL)	-	-	-	296.11
Luteinizing hormone (RV 1.14-8.75 mUI/mL)	-	-	-	8.36
Follicle stimulating hormone (RV 1.37-13.58 mUI/mL)	-	-	-	6.74
Growth hormone (RV 3 ng/mL ng/mL)	-	-	-	6.76
Prolactin (RV 2.58-18.12 ng/mL)	-	-	-	18.48
Basal cortisol (RV 3.7-19.4 μg/dL)	-	-	-	33.4
Viral serologies for hepatitis B. C and HIV	Negative	-	-	Negative

RV: reference value.

**Figure 1:**
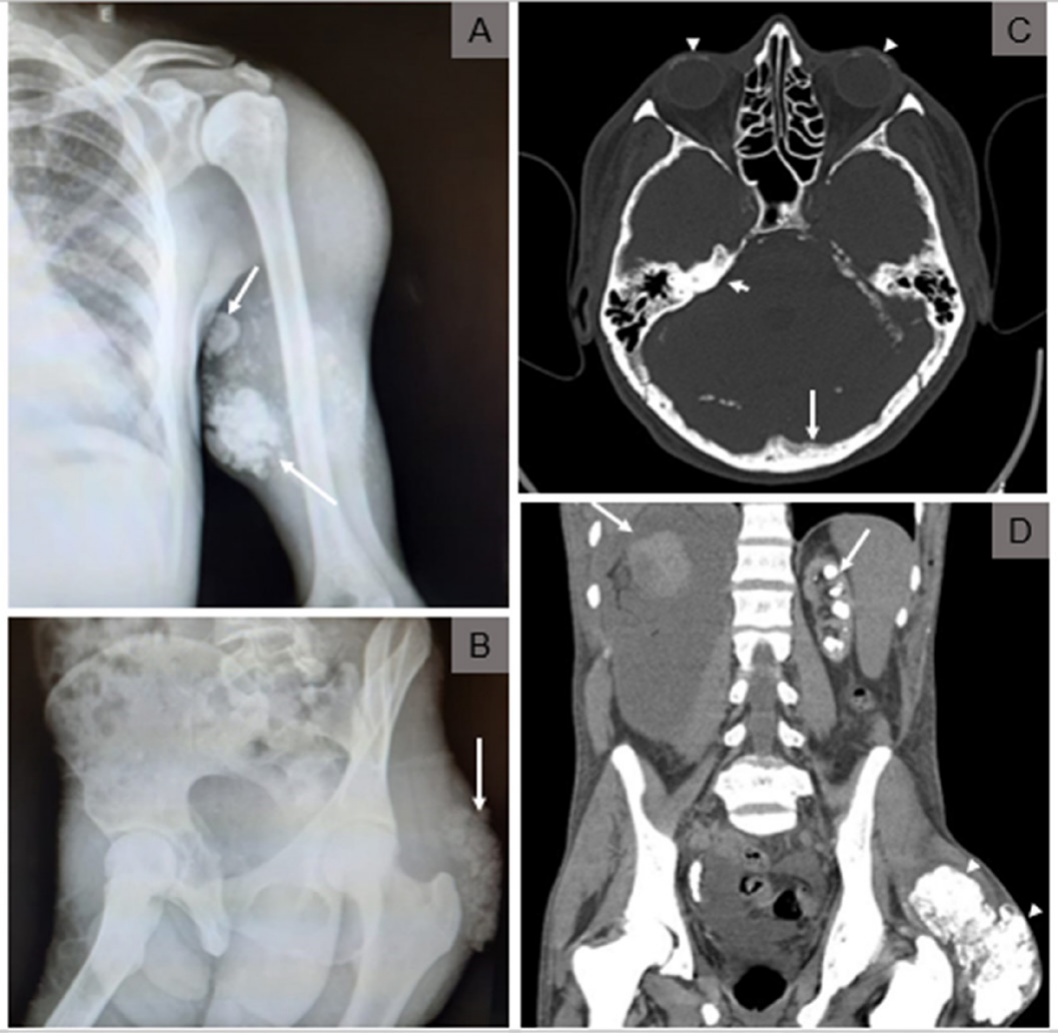
A and B: X-Ray imaging of frequent oil injection sites show triceps (A) and gluteus (B) calcifications (arrows). (C) Head computerized tomography imaging show: inner ear and sclera calcifications (arrow heads), meningeal dura mater calcifications (long arrow); (D) Abdominal CT showing nephrocalcinosis bilaterally, left kidney atrophy, right hydronephrosis (long arrow), and gluteus calcifications (arrow heads).

**Figure 2:**
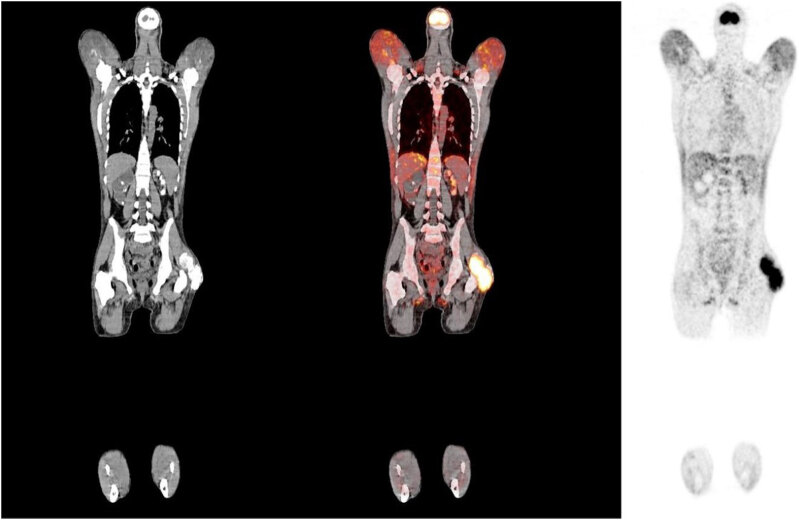
Whole-body 18F-FDG-PET-CT (18-fluor-deoxy-2-glucose computerized tomography) showing extensive increased metabolic activity in the glutei regions, biceps, triceps, brachii, deltoids, pectoralis major, trapezius, and latissimus dorsi, and images of diffuse calcinosis more evident in the eyeballs, meninges, heart valves, lungs, kidneys, various subcutaneous projections, and muscle planes.

Computerized tomography of the skull, chest, and abdomen revealed bilateral peri cochlear density and meningeal calcifications in the dura mater and sickle region; pleural calcification and nonspecific interstitial pneumonitis; calcification of the renal parenchyma suggestive of nephrocalcinosis bilaterally, in addition of left kidney atrophy and right hydronephrosis (Figure 1). Transthoracic echocardiogram showed atrial and right ventricular dilation, concentric left ventricular hypertrophy, and mitral and aortic valve calcification. A whole-body 18F-FDG-PET-CT (18-fluor-deoxy-2-glucose computerized tomography) showed extensive and symmetric increased metabolic activity in the glutei regions, biceps, triceps brachii, deltoids, pectoralis major, trapezius, and latissimus dorsi, as shown in Figure 2. It also exhibited images of diffuse calcinosis more evident in the eyeballs, meninges, heart valves, lungs, kidneys, various subcutaneous projections, and muscle planes.

The patient was diagnosed with PTH-independent hypercalcemia, due to 1,25(OH)D production in hyperactive connective tissue and foreign-body granulomas, with extensive multiple organs and soft tissue calcifications, peripheral facial paralysis due to calcifications on the neural path, and body dysmorphic disorder. He was initially managed with hemodialysis three times a week, hydrocortisone 200 mg intravenously for 8 days until discharge, and zoledronic acid 2 mg intravenously was given once. Additional therapy was prescribed by psychiatry with sertraline 50 mg (p.o.) and quetiapine 50 mg (p.o.). The patient was discharged receiving prednisone 20 mg (p.o.). His calcium levels gradually decreased to 10.4 mg/dL after 10 days of hospitalization.

## DISCUSSION

In this report, a patient with PTH-independent hypercalcemia was described. He presented with nonspecific symptoms, such as longtime asthenia, frequent vomiting, and recurrent nephrolithiasis, that can be compatible with chronic hypercalcemia.

The patient also evolved with renal failure and hemodialysis, which are severe complications of chronic hypercalcemia (3).

The most common non-parathyroid hypercalcemia cause is malignancy, frequently after skeletal metastasis or by the production and release of hypercalcemic factors such as PTH-related peptide (PTH-rP), which can bind PTH receptors (14). Tumors causing hypercalcemia are mostly advanced and metastatic diseases (3). Our patient showed no signs and symptoms of malignancy neither during clinical evaluation nor during laboratory and imaging examination, thus this hypothesis was excluded. PTH-rP was not accessed due to its unavailability in our unit.

Endocrinopathies other than hyperparathyroidism have also been associated with mild hypercalcemia, notably hyperthyroidism, due to increased bone turnover, and adrenal insufficiency, due to increased calcium flux into the extracellular space, as well as volume depletion (14). The present patient had no thyroid or adrenal dysfunctions.

Vitamin D-mediated hypercalcemia normally presents with low PTH levels and might be most commonly caused by exogenous vitamin D toxicity or excessive production of vitamin D metabolites (2). Our patient had a history of intramuscular injection of an oily veterinary product composed of vitamins A, D, and E, however, his current levels of 25-hydroxyvitamin D3 were normal to low, and the time of onset of the symptoms was incompatible since in vitamin D intoxication they tend to be more acute (12,14). There is one series of cases reporting 16 patients that evolved with hypercalcemia secondary to the same intramuscular injectable supplements and the onset of symptoms was also inconsistent (12), supporting that there is probably another mechanism behind it in addition to the absorption of the injected vitamin D.

A recently described etiology of vitamin D-mediated hypercalcemia is a foreign body reaction due to silicone, paraffin, and other oil compounds injections associated with cosmetic procedures (15). The overactivity of extrarenal 1-alpha-hydroxylase in activated macrophages in granulomas is believed to lead to the pathological 1,25(OH)D (calcitriol) production and subsequent hypercalcemia (7). There are cases described in the literature of patients who made use of injectable fillers, such as silicone, mineral oil, paraffin, and polymethylmethacrylate, for aesthetic reasons and later developed hypercalcemia (8,10,11,16,17). The clinical condition is frequently diagnosed years after initial cosmetic injection and can be severe enough to cause renal failure and death (7). Patients tend to present with diffuse subcutaneous calcifications and hardening of the muscles involved (10,11,17). Similar to our patient's laboratory findings, most cases present with elevated calcitriol levels, suppressed PTH, and low/normal 25-hydroxyvitamin D levels (7).

FDG PET-CT imaging from other similar reports showed increased metabolic activity in exact muscle groups described to be injection sites, just as in the case (8,10,18). An important differential diagnosis is sarcoidosis, as it is also a granulomatous disease that can develop with PTH-independent hypercalcemia. Nevertheless, our patient had an inconclusive clinical picture for sarcoidosis, in addition to non-significant mediastinal lymph node and lung involvement in FDG PET-CT, which are affected in more than 90% of patients with sarcoidosis (19).

It is important to highlight that our patient was a chronic androgenic-anabolic steroids user, and these substances are known for causing glomerular and interstitial damage by direct renal toxicity, glomerular hyperfiltration, and hypercalcemia (20). Our patient also administered high doses of vitamin A (greater than 50,000 IU/day), which have been associated with hypercalcemia due to enhanced bone resorption (3,12). Thus, the mechanisms of hypercalcemia and renal failure related to the case presented are probably multifactorial, with some more relevant than others.

A trial of corticosteroid therapy has been widely advocated in the acute management of hypercalcemia associated with high levels of calcitriol, originating from an extrarenal source (7,11). Other less relevant treatment options are bisphosphonates and denosumab and even less often calcitonin and ketoconazole were used due to resistant hypercalcemia (7). Patients tend to evolve with calcium normalization in days to months, however, a good portion of them may relapse (7). The case presented was treated with corticosteroid and zoledronic acid and had little significant response, possibly due to the short follow-up. Some authors may advocate the excision of the paraffin lesions as a treatment option (7,14), but in our case there were many extensive multisystemic paraffin lesions, rendering the surgical intervention impossible and even dangerous.

We report a case of a young bodybuilder who developed PTH-independent hypercalcemia caused by foreign body granulomas in the areas of oil injection and evolved with serious and irreversible complications of chronic hypercalcemia. The medical community must be aware of all possible complications associated with surreptitious aesthetic procedures to educate the general population. Physicians also must be concerned about underlying psychiatric diseases leading to such behaviors, otherwise, patient's treatment compliance will not be accomplished.
